# Salivary MicroRNAs as Potential Noninvasive Biomarkers for the Diagnosis of Nasopharyngeal Carcinoma: Protocol for a Scoping Review

**DOI:** 10.2196/69484

**Published:** 2025-07-04

**Authors:** Anton Sony Wibowo, Sagung Rai Indrasari, Camelia Herdini, Dewajani Purnomosari, Sulis Ernawati

**Affiliations:** 1 Doctoral Program, Faculty of Medicine, Public Health, and Nursing School of Medicine Universitas Gadjah Mada Yogyakarta Indonesia; 2 Academic Hospital Universitas Gadjah Mada Yogyakarta Indonesia; 3 Otorhinolaryngology-Head and Neck Surgery Department Faculty of Medicine, Public Health, and Nursing, School of Medicine Universitas Gadjah Mada Yogyakarta Indonesia; 4 Department of Histology and Cell Biology Faculty of Medicine, Public Health, and Nursing, School of Medicine Universitas Gadjah Mada Yogyakarta Indonesia

**Keywords:** nasopharyngeal carcinoma, microRNA, miRNA, biomarker, saliva, diagnosis

## Abstract

**Background:**

Nasopharyngeal carcinoma (NPC) is the fourth-most-prevalent cancer in both Indonesia and Asia. Globally, an estimated 133,354 cases and 80,008 deaths were attributed to NPC in 2020. Early diagnosis plays a key role in managing NPC. Molecules found in bodily fluids, such as saliva, contain compounds (including microRNAs [miRNAs]) that can aid in detecting diseases like NPC. More studies on the expression, role, use, and accuracy of salivary miRNAs as potential diagnostic biomarkers of NPC are needed.

**Objective:**

This protocol provides a framework for conducting a scoping review aimed at mapping the expression, role, use, and diagnostic accuracy of specific salivary miRNAs as potential diagnostic biomarkers of NPC.

**Methods:**

The guidelines established by the JBI will be followed, which include defining the research questions, identifying relevant studies, and selecting studies based on titles and abstracts. The process will involve charting the data; collating, summarizing, and reporting the findings; and consultation. The synthesis will specifically examine the expression, role, use and accuracy of salivary miRNAs as potential diagnostic biomarkers of NPC. Both quantitative and qualitative analyses will be performed.

**Results:**

The selection of studies based on keywords was completed in August 2024, and the study screening and review processes were finished by November 2024. The drafting of the scoping review manuscript is currently underway. The development of this scoping review protocol was supported by funding from the Centre for Higher Education Funding and the Indonesia Endowment Fund for Education.

**Conclusions:**

The findings of this study will offer a comprehensive overview of the expression, role, use, and diagnostic accuracy of salivary miRNAs as potential diagnostic biomarkers of NPC. The findings of this review are expected to serve as a basis for further research.

**Trial Registration:**

Open Science Framework k3prh_v1; https://osf.io/preprints/osf/k3prh_v1

**International Registered Report Identifier (IRRID):**

DERR1-10.2196/69484

## Introduction

### Background

Nasopharyngeal carcinoma (NPC) is a malignant cancer that arises from the epithelium of the nasopharynx. The global incidence of NPC is rising; despite this, mortality rates and disability-adjusted life years are decreasing. The heaviest burden of NPC is in regions with a high-middle or middle sociodemographic index [1]. This cancer is prevalent in Indonesia, southern China, Southeast Asia, and North Africa [2]. Indonesia ranks as the third-highest country in terms of mortality due to NPC, reaching 3220 deaths in 2019 [3].

Diagnosing NPC is challenging. The nasopharynx is in the postnasal space, which is difficult to access with simple examinations. Additionally, the nasopharynx often has a normal lymphoid epithelium that is hard to distinguish from NPC. Clinical appearances of NPC are often nonspecific, leading to missed or delayed diagnoses [4]. Survival rates have not significantly improved in recent decades. This is due to the majority of cases being diagnosed at advanced clinical stages and the high treatment failure rates associated with late diagnosis [5]. Treatment for NPC at an early stage has a much better prognosis compared to the advanced stages [6]. Therefore, early detection is crucial in managing NPC [7].

MicroRNAs (miRNAs) are small molecules consisting of nucleotides with a single strand. These small RNA molecules regulate protein production from messenger RNA (mRNA) [8]. Research has reported that changes in miRNA expression are closely related to carcinogenesis, including in NPC. These molecules play a role in the transformation of normal epithelial cells into neoplastic cells [9]. miRNAs contribute to the increased migration and invasion of NPC cells by upregulating messenger RNA that encodes extracellular matrix proteins. Certain miRNA alterations also downregulate the expression of several tumor suppressors, and they affect cell proliferation, colony formation, migration, and invasion in vitro [10].

Saliva is an easily accessible bodily fluid and one example of a noninvasive sample that has the potential for biomarker detection in NPC. Saliva contains more than 3000 types of RNA that can be used as biomarkers for the noninvasive diagnosis of NPC [7]. Therefore, the molecules contained in saliva can potentially be used to detect several diseases, including NPC. [11]

Most previous studies have explored alternative methods for the detection of NPC. These investigations and reviews have primarily focused on tissue biopsy, various molecular markers found in tissue samples, and other invasive techniques [12-19]. There is no comprehensive review that has specifically examined and combined the use of salivary miRNAs as a noninvasive modality for NPC detection. Our scoping review aims to bridge this gap by providing an up-to-date synthesis of current evidence and identifying new areas in using salivary miRNAs as noninvasive tools for diagnosing NPC.

Early detection remains a clinical challenge due to the deep anatomical location of NPC and the nonspecific initial symptoms. While tissue biopsy remains the gold standard for diagnosis, it is invasive, often delayed, and limited by accessibility and patient compliance. These limitations point to the urgent need for noninvasive, reliable diagnostic alternatives. Recent advances in molecular oncology have highlighted the potential of miRNAs as promising noninvasive diagnostic biomarkers of NPC. Among various bodily fluids, saliva has emerged as an attractive medium for biomarker discovery due to its noninvasive collection and ease of storage. Several studies have started to look at the mechanisms underlying new diagnostic methods for NPC, but most of the results still mainly focus on tissue biopsy and other methods that are more invasive, and there is no comprehensive overview of the findings.

To address this gap and provide a comprehensive overview of the current landscape, this scoping review will explore the following topics:

- Mapping the expression of miRNA in the saliva of patients with NPC
- Mapping the role, use, and accuracy of specific salivary miRNAs as potential noninvasive diagnostic biomarkers for patients with NPC

### Objectives

This protocol is designed to guide a scoping review by outlining research objectives, methods, study selection, and data extraction. The objectives of this scoping review protocol are as follows:

- Identify and understand the expression of salivary miRNAs in patients with NPC
- Understand the role of specific miRNAs in NPC as potential noninvasive diagnostic biomarkers of NPC.

## Methods

### Overview

This scoping review will be carried out in accordance with the JBI scoping review methodology [20]. There are six steps: (1) define the research questions; (2) identify relevant studies; (3) select the studies; (4) chart the data; (5) collate, summarize, and report the results; and (6) consult [21]. The PRISMA-ScR (Preferred Reporting Items for Systematic reviews and Meta-Analyses Extension for Scoping Reviews) checklist will be used [22]. Searching the literature, refining the search strategy, assessing articles for inclusion, and collecting pertinent data will be done in an iterative fashion.

### Protocol Registration and Report Information

A PRISMA-ScR checklist is provided in Multimedia Appendix 1. The checklist is an effective tool for guiding the reporting of scoping studies [23]. This protocol was registered on the Open Science Framework (k3prh_v1) on April 2, 2024 [24].

### Research Questions

The research questions are as follows:

1. What is the expression of salivary miRNAs in patients with NPC?
2. What are the roles, uses, and diagnostic accuracy of specific salivary miRNAs as potential diagnostic biomarkers of NPC?

### Eligibility Criteria

The research questions and eligibility criteria were designed following the population, concept, context framework (Multimedia Appendix 2). This approach supports researchers in defining the core elements of a scoping review, generating relevant search terms to investigate the topic, and choosing specific inclusion and exclusion criteria.

#### Population

The population of interest [25] includes patients suspected of having NPC and showing symptoms and signs of the condition but without a definitive diagnosis. For example, this could include patients with a neck lump who have not undergone a biopsy. It also includes patients from regions with a high incidence of NPC who have undergone screening to detect the disease [26].

#### Concept

Studies were included that investigated the diagnostic potential, biological roles, and expression patterns of salivary miRNAs as noninvasive biomarkers (ie, liquid biopsies) of NPC. This includes studies assessing their diagnostic accuracy, applicability, and clinical utility.

#### Context

We included research conducted in clinical or laboratory settings related to the early detection or diagnosis of NPC using salivary miRNAs.

### Identifying Relevant Studies

Stage 2 of the review had 2 steps. The first involved identifying all relevant scoping reviews published up to March 2024. The search was performed in the Cochrane and Scopus databases without any time restrictions. No scoping reviews were identified focusing on salivary miRNAs as biomarkers for NPC diagnosis. The second step was to identify research on the expression of salivary miRNAs and the roles, uses, and diagnostic accuracy of specific salivary miRNAs as potential diagnostic biomarkers of NPC.

### Search Strategy

A systematic search for relevant studies was conducted in 8 databases: PubMed, Google Scholar, BMJ Journal, ClinicalKey, EBSCOhost, Nature, SpringerLink, and Scopus. Gray literature was explored through GreyNet International. The selected journal databases were chosen based on their reputable indexing and accessibility from our institution, as our institution maintains subscriptions to these databases. We included Google Scholar to supplement our primary database search by identifying gray literature, preprints, and emerging studies that may not be indexed in conventional databases, which is especially useful for a developing research topic like salivary miRNAs in NPC. Additionally, manual searches were performed by reviewing references from eligible articles and other pertinent publications. All search results and the screening process were transferred to Rayyan, a free online tool for managing systematic reviews. Articles meeting the inclusion criteria were selected for inclusion (Multimedia Appendix 3). The authors used Medical Subject Headings (MeSH) terms to perform database searches with keywords associated with NPC, miRNAs, biomarkers, and saliva (Multimedia Appendix 4). The literature search strategy adhered to the PRESS (Peer Review of Electronic Search Strategies) guidelines, covering 6 essential stages: translation; use of Boolean and proximity operators; subject headings; text word searches; checking spelling, syntax, and line numbers; and applying limits and filters [27]. The search strategy was based on key elements from the PICO (population, intervention, comparator, outcome) framework to generate effective search terms [22].

### Study Selection

The search results from stage 2 were transferred to Rayyan for data management, including duplicate removal and referencing. Duplicate articles were eliminated [28]. Three independent reviewers screened the titles, abstracts, and full texts according to the inclusion and exclusion criteria. Studies meeting the inclusion criteria continued to full-text screening, while those that did not were excluded. The reasons for exclusion will be detailed in the final report. Two authors conducted separate literature searches, and their results were then compared. Any discrepancies were resolved through discussion or by consulting a third researcher if necessary. Data extraction was conducted by one investigator, while a second investigator independently reviewed the data to ensure completeness and accuracy. The final selection of papers and the relevant data related to our primary question will be summarized in a PRISMA-ScR diagram [22].

### Charting (Extracting) the Data

#### Data Extraction

Three reviewers were involved in the data extraction process to identify and discuss eligible articles for this review. Two reviewers independently conducted the literature search, and their results were compared. Any disagreements were resolved through discussion or, if needed, with the involvement of a third reviewer to make the final decision. One investigator extracted the data, while a second independently verified it for accuracy and completeness. Three experienced reviewers validated the search results. A Microsoft Excel sheet will be prepared to extract key information from the selected studies (Multimedia Appendix 5).

#### Data Synthesis

The JBI scoping review guideline will be used for data synthesis [29]. This synthesis focuses on the expression, role, use, and diagnostic accuracy of salivary miRNAs as potential diagnostic biomarkers of NPC. Quantitative analysis will be presented descriptively in tables, frequency distribution tables, and statistical summaries. Qualitative analysis will be presented in a narrative format to describe the expression of specific salivary miRNAs in patients with NPC and the role of specific miRNAs in NPC as potential diagnostic biomarkers.

We gathered key details from the included studies, such as citation information, country of origin, objectives, population, sample size, sample attributes, study methods, laboratory techniques used (eg, miRNA microarray data, reverse transcription quantitative polymerase chain reaction, the Cancer Genome Atlas), type of intervention, comparator details (including the number and characteristics of comparators), duration, outcomes, and main findings relevant to the expression, roles, uses, and diagnostic accuracy of salivary miRNAs as potential diagnostic biomarkers (ie, the review objective). Two researchers will review and validate the results of the data analysis. Any discrepancies will be addressed and resolved collaboratively.

#### Collating, Summarizing, and Reporting the Results

The relevant references identified in the scoping review will be imported into Mendeley, with database listings exported in RIS format, including full-text references. A PRISMA-ScR flow diagram will be used to illustrate the stages of the literature screening process for articles deemed eligible for analysis. Descriptive analysis and tabulation will be used for a narrative summarization.

Frequencies and percentages will be used to describe nominal data. The results will be presented and organized into nine main sections: (1) year of study, (2) country, (3) research objectives, (4) research methods, (5) examination techniques used, (6) population, (7) sample size, (8) comparators, and (9) key findings related to miRNAs and their relationship with the pathogenesis and diagnosis of NPC.

### Consultation

Consultation can enhance the results and substantially strengthen scoping review processes and outcomes [30]. The research team will engage in discussions with consultants to review and refine the initial findings, working collaboratively to achieve a consensus. Consultation with experts in the field will be conducted after the initial data charting and initial analysis to summarize the data, validate the findings, correct conceptual errors, and enhance the interpretation of results. Biomolecular experts will be consulted if additional data or information are required.

### Ethical Considerations

This review will not involve humans, animals, patients, or the public. It will compile findings from studies that have already been peer-reviewed and published. As a result, ethical clearance is not required.

## Results

The selection of studies based on keywords was completed in August 2024. The study screening and review processes were finalized by November 2024. The drafting of the scoping review manuscript is currently underway, with the review expected to be completed by the end of August 2025.

This paper will highlight key findings, including study selection criteria; characteristics of the included studies; an overview of the expression, role, use, and diagnostic accuracy of salivary miRNAs as potential diagnostic biomarkers for NPC; a critical evaluation of strengths and limitations; and future research recommendations.

The scoping review is funded by the Ministry of Education through the Indonesian Educator Scholarship program (2022-2025), with no involvement from the funders in the development of the review protocol.

## Discussion

### Principal Findings

Early detection of NPC is crucial for improving treatment outcomes and enhancing patient prognoses. Researchers have shown that early identification of NPC leads to better treatment results, increased survival rates, and lower chances of recurrence [26].

The primary diagnostic methods for NPC rely on tissue biopsy, which is considered the gold standard. However, tissue biopsy has several limitations, as it is an invasive procedure for patients, it is not suitable for long-term monitoring due to a lack of sufficient data on the spatial and temporal variability of the tumor [13], and it can be affected by patient compliance. This highlights the importance of this scoping review protocol for identifying alternative detection methods for the future that offer comparable accuracy to tissue biopsy while being less invasive and more accessible. Thus, while tissue biopsy remains the diagnostic reference standard, our review explores the potential of salivary miRNAs as a complementary and future noninvasive tool that may overcome some of the limitations of tissue biopsy.

Saliva is a readily accessible bodily fluid and may serve as a noninvasive potential source of biomarkers, such as miRNAs, for the detection of NPC [7,26]. The use of salivary miRNAs is recognized as a noninvasive and cost-effective modality for the detection of cancers such as NPC. Saliva offers advantages in collection, transportation, and processing compared to blood or tissue samples [31].

Protocols are widely considered a crucial element in enhancing the value of research and minimizing waste [29]. Scoping review protocols are crucial, as they provide a structured framework for reviewers on the direction and implementation of the review process. A protocol can be seen as a methodological guide that outlines key components, such as inclusion criteria, the databases and search strategies for identifying studies, and the overall methodological approach [29,32]. It is strongly advised to establish protocols prior to starting a scoping review [33].

This review is expected to encounter several difficulties. This topic is a new field. The availability of articles discussing miRNAs as potential diagnostic biomarkers of NPC is still quite limited. To address this issue, the researchers plan to broaden the range of article databases used. The researchers will expand the types of studies included in this review. The novelty of this topic can also be seen as one of the advantages of this review. Another potential difficulty is that the medical terminology used in each article for miRNAs, NPC, and biomarkers varies greatly. To mitigate this, we will use specific keywords.

The findings of this study will offer a comprehensive overview of the expression, role, use, and diagnostic accuracy of salivary miRNAs as potential noninvasive diagnostic biomarkers of NPC. It is well established that noninvasive biomarkers of cancer can improve the quality of cancer treatment [6]. This scoping review focuses solely on the role of saliva miRNAs as potential biomarkers for the diagnosis of NPC. The findings of this review are expected to serve as a basis for further research.

Our plan for dissemination involves delivering presentations at international conferences, as well as publishing in a peer-reviewed journal. A limitation of this study is that diagnosing NPC cannot depend solely on potential biomarkers like miRNAs as the main method. Incorporating other diagnostic techniques, such as radiological imaging, will still be needed to improve the accuracy of NPC diagnosis in future research. Incorporating these variables would enable a more comprehensive analysis.

### Conclusion

This scoping review protocol will provide a comprehensive overview of the existing literature on the expression of specific salivary miRNAs in patients with NPC and will map the role, use, and accuracy of miRNAs in NPC as potential diagnostic biomarkers.

## Appendix

Multimedia Appendix 1 PRISMA-ScR (Preferred reporting items for systematic reviews and meta-analyses protocols Extension for Scoping Reviews) checklist.

Multimedia Appendix 2 PICO (population, intervention, comparator, outcome) framework.

Multimedia Appendix 3 Inclusion and exclusion criteria.

Multimedia Appendix 4 Search strategy.

Multimedia Appendix 5 Data extraction variables.

## Abbreviations

MeSH: Medical Subject Headings
miRNA: microRNA
NPC: nasopharyngeal carcinoma
PICO: population, intervention, comparator, outcome
PRESS: Peer Review of Electronic Search Strategies
PRISMA-ScR: Preferred Reporting Items for Systematic Reviews and Meta-Analyses Extension for Scoping Reviews
