# Supplementary material for: Salivary MicroRNAs as Potential Noninvasive Biomarkers for the Diagnosis of Nasopharyngeal Carcinoma: Protocol for a Scoping Review
Source: JMIR Res Protoc. 2025 Jul 4;14:e69484. doi: 10.2196/69484 (PMC12274780; doi:10.2196/69484)
Supplement: Multimedia Appendix 2 [file resprot_v14i1e69484_app2.docx]

**Multimedia Appendix 2**

**PICO Framework**

| **Participants/Population** | Individuals suspected of having nasopharyngeal carcinoma or populations at risk of developing nasopharyngeal carcinoma |
| --- | --- |
| **Concept** | The expressions, biological roles, diagnostic utility, and diagnostic accuracy of salivary miRNAs as potential non-invasive diagnostic biomarkers.  The use of salivary miRNAs as non-invasive biomarkers (liquid biopsy) for the detection or diagnosis of nasopharyngeal carcinoma. |
| **Context** | Studies conducted in clinical or laboratory settings focused on the early detection or diagnosis of nasopharyngeal carcinoma using salivary miRNAs. |
