# Supplementary material for: Salivary MicroRNAs as Potential Noninvasive Biomarkers for the Diagnosis of Nasopharyngeal Carcinoma: Protocol for a Scoping Review
Source: JMIR Res Protoc. 2025 Jul 4;14:e69484. doi: 10.2196/69484 (PMC12274780; doi:10.2196/69484)
Supplement: Multimedia Appendix 3 [file resprot_v14i1e69484_app3.docx]

**Multimedia Appendix 3**

Inclusion and Exclusion Criteria

| Inclusion Criteria | Exclusion Criteria |
| --- | --- |
| - Human subjects - microRNA from body fluids and tissues - original article - Articles are written in English or Indonesian - Presented in the form of qualitative or/and quantitative data. - The article has an IMRAD component (introduction material and method, results, discussions) - The search strategy is not limited by publication date. All articles up to 3 May 2023, which met the inclusion criteria were assessed. | - Studies with subject patients with incident malignancies/tumors other than malignancies/tumors of the head and neck area - The diagnosis of cancer is made not with anatomical pathology justification. - Letters, narrative reviews, and animal studies |
