# Supplementary material for: Salivary MicroRNAs as Potential Noninvasive Biomarkers for the Diagnosis of Nasopharyngeal Carcinoma: Protocol for a Scoping Review
Source: JMIR Res Protoc. 2025 Jul 4;14:e69484. doi: 10.2196/69484 (PMC12274780; doi:10.2196/69484)
Supplement: Multimedia Appendix 4 [file resprot_v14i1e69484_app4.docx]

**Multimedia Appendix 4**

Search Strategy

Search Terms (Mesh Term)

**Selected databases:** PubMed, Google scholar, BMJ journal, Clinical key, EBSCO host, Nature, SpringerLink, and Scopus

MeSH ((((((((((("Body Fluid"[Mesh]) OR "Circulating"[Mesh]) OR "Saliva"[Mesh]) OR " Biomarker "[Mesh]) OR " Nasopharyngeal Carcinoma "[Mesh]) OR " Cancer of Nasopharynx "[Mesh]) OR " Nasopharyngeal Neoplasm "[Mesh]) AND "miRNA"[Mesh]) OR "mirn"[Mesh]) OR "mir"[Mesh])

Search terms

**Published date**: All Years

**Resource types**: Articles
