# Supplementary material for: Salivary MicroRNAs as Potential Noninvasive Biomarkers for the Diagnosis of Nasopharyngeal Carcinoma: Protocol for a Scoping Review
Source: JMIR Res Protoc. 2025 Jul 4;14:e69484. doi: 10.2196/69484 (PMC12274780; doi:10.2196/69484)
Supplement: Multimedia Appendix 5 [file resprot_v14i1e69484_app5.docx]

**Multimedia Appendix 5**

Data Extraction Variables

| **Variable** | **Description** |
| --- | --- |
| Study details | Author, date/years, title, journal, volume, issue, pages, country, objectives of study, and design study |
| Objectives | Specific statements that describe the aim of the study |
| **Variables relating to process/method** | |
| Participants demographics | Country, Age, gender, others relevant demographics variable |
| Methodology used | Experimental  Observational analitic  (type of intervention, comparison, duration of study, sample size, data collections) |
| **Variable relating to research** | |
| type of sample | Type of body fluid was used as source of miRNA |
| type of examination | Methode of miRNA examnation |
| type of intervention | Specific miRNA which was used as nasopharyngeal carcinoma biomarker |
| Role, use and accuracy of miRNA related to nasopharyngeal carcinoma detection. | Feasibility and accuracy use of miRNA related to nasopharyngeal carcinoma detection ( diagnostic value of miRNA related to nasopharyngeal carcinoma detection) |
| Assessment process (author reflections) | Reported results, strengths, limitations, and recommendation, others important findings related to the aim of the review |
